# Enhanced oral bioavailability of koumine by complexation with hydroxypropyl-β-cyclodextrin: preparation, optimization, *ex vivo* and *in vivo* characterization

**DOI:** 10.1080/10717544.2021.1998248

**Published:** 2021-11-12

**Authors:** Qing Hu, Xiaoling Fu, Yanping Su, Yanfang Wang, Sihuan Gao, Xiaoqin Wang, Ying Xu, Changxi Yu

**Affiliations:** a School of Pharmacy, Fujian Medical University, Fuzhou, China; b Fujian Key Laboratory of Drug Target Discovery and Structural and Functional Research, School of Pharmacy, Fujian Medical University, Fuzhou, China

**Keywords:** Koumine, hydroxypropyl-β-cyclodextrin, inclusion complexes, permeability, bioavailability

## Abstract

Koumine (KME) is an active alkaloid extracted from *Gelsemium elegans*, and its diverse bioactivities have been studied for decades. However, KME exhibits poor solubility and low oral bioavailability, which hampers its potential therapeutic exploitation. This work aimed to develop optimized inclusion complexes to improve the bioavailability of KME. The KME/hydroxypropyl-β-cyclodextrin (KME/HP-β-CD) inclusion complexes were prepared by the solvent evaporation method and later optimized using the Box-Behnken design. The optimal KME/HP-β-CD was characterized by scanning electron microscopy, Fourier transforms infrared spectroscopy, differential scanning calorimetry, and nuclear magnetic resonance spectroscopy. The physicochemical characterization results revealed that the crystalline state of KME was transformed into an amorphous form, forming KME/HP-β-CD inclusion complexes. Compared with KME, the solubility and *in vitro* release rate of KME/HP-β-CD was significantly enhanced by 52.34- and 1.3-fold, respectively. Further research was performed to investigate the intestinal absorption characteristics and *in vivo* bioavailability in rats. The optimal KME/HP-β-CD showed enhanced absorptive permeability and relative bioavailability increased more than two-fold compared to that of raw KME. These results indicate that the optimal KME/HP-β-CD can be used as an effective drug carrier to improve the solubility, intestinal absorption, and bioavailability of KME.

## 1. Introduction

*Gelsemium elegans* Benth. (*G. elegans*), a plant native to China and Southeast Asia is widely used in Chinese folk medicine to treat inflammatory diseases and pain (Yang et al. 2016). To date, more than 100 alkaloids have been isolated from *G. elegans*, and they are regarded as the most likely active groups responsible for the observed pharmacological effects of this plant. Among these alkaloids, koumine (KME) is the most abundant and exhibits a diverse set of biological effects, including antitumor, analgesic, anti-inflammatory, anxiolytic, and protection from neurotropic disorders (Jin et al. 2014; Zhang & Wang 2015; Yuan et al. 2016). An efficient and simple method for separating, extracting, and purifying KME from *G. elegans* was established by our research team (Su et al. 2011). Moreover, over the past fourteen years, we have conducted several studies to evaluate the pharmacological effects and toxicity of KME (Ming et al. 2011; Qiu et al. 2015; Yu et al. 2015). An invention patent for the application of KME for anti-inflammatory therapy has been authorized by China and the United States (US 9078890 B2). Currently, there is an urgent need to obtain a preclinical basis for a simple and robust oral dosage form of KME. However, it is well known that the pharmacokinetic properties of many natural medicinal ingredients are limited due to their solubility (Raza et al. 2017). KME is a lipophilic molecule with poor solubility in water (<1 mg/mL). The intrinsic low solubility and poor oral bioavailability properties of KME severely restrict its clinical applications. Therefore, it is necessary to adopt formulation technology to improve its solubility and bioavailability.

The clinically preferred route of drug delivery is oral administration. The main purpose of the oral drug delivery system is to modulate the solubility of the active pharmaceutical ingredient (API), thereby improving its absorption and bioavailability. Most newly developed APIs are lipophilic with low aqueous solubility and poor absorption, resulting in low bioavailability, which will lead to therapeutic failure. To enhance solubility and dissolution, pharmaceutical researchers have used various approaches (Khalid et al. 2018; Vikas et al. 2018; Giri et al. 2021), such as particle size reduction, prodrug, solid dispersions, cyclodextrin (CD) complex, self-emulsifying, and salt formation. These methods each have their own advantages and disadvantages, depending on the particular drug, polymer, properties, stability, technology transfer from the laboratory to the industrial scale, *etc.* (Garcia-Otero et al. 2021). Among these, inclusion complexes with CD have been generally used to increase the oral bioavailability of poorly aqueous solubility drugs (Hu et al. 2019; Khalid et al. 2021). CD is characterized by a hydrophobic interior cavity that can accommodate various lipophilic drugs and a hydrophilic exterior surface. Several CD derivatives such as hydroxypropyl, sulfobutyl ether, methyl, and dimethyl derivatives have high water solubility and are widely used in the field of medicine. An extensive literature survey shows that hydroxypropyl-β-cyclodextrin (HP-β-CD) is the safest among all CD derivatives because it does not absorb through biological membranes (Al-Heibshy et al. 2020; Manta et al. 2020). In addition, HP-β-CD was the first β-CD derivative to be approved by the FDA for both oral and intravenous administration (Raza et al. 2017).

To our knowledge, our work is the first report to describe the complexation of KME with HP-β-CD (KME/HP-β-CD) to improve its water solubility and bioavailability. The novelty of this work is its provision of a preclinical basis for oral KME dosage forms that are easy to prepare and scalable from the laboratory scale to the industrial scale. In addition to water solubility, the permeability of drugs through biological membranes is regarded as the second most important parameter for determining the oral bioavailability of active agents (Rong et al. 2013; Shankar et al. 2021). Hence, the present work has not only studied the enhanced water solubility of KME but also its permeability characteristics in everted gut sacs and Caco-2 cell models. In this study, the process parameters of KME/hydroxypropyl-β-cyclodextrin inclusion complexes (KME/HP-β-CD) were optimized by a Box-Behnken design (BBD) to obtain the highest complexation efficiency. The optimal KME/HP-β-CD was prepared and characterized by scanning electron microscopy (SEM), differential scanning calorimetry (DSC), powder X-ray diffraction (PXRD), Fourier transform infrared (FT-IR) spectroscopy, and ^1^H nuclear magnetic resonance (^1^H NMR). Furthermore, *ex vivo* permeation studies were performed to evaluate the effects of HP-β-CD on the permeation of KME across rat intestines and Caco-2 cell monolayers. The oral bioavailability of KME and KME/HP-β-CD was evaluated in rats.

## 2. Materials and methods

### 2.1. Materials

Koumine was isolated from *G. elegans* by one of the authors (Yanping Su) using the method reported earlier (Su et al. 2011) and the purity reached 99.0%. Hydroxypropyl-β-cyclodextrin (HP-β-CD) was a gift sample from Shandong Qianhui Biotechnology Co. Ltd., China. Dulbecco’s modified Eagle medium (DMEM) and fetal bovine serum (FBS) were purchased from HyClone, USA. HBSS buffer was purchased from Shanghai Baisai Media Technologies Co. Ltd., China. Sodium chloride, potassium chloride, calcium chloride, sodium bicarbonate, potassium dihydrogen phosphate, glucose, and magnesium chloride were all obtained from Sinopharm Reagents Co. Ltd., China. All organic solvents were of analytical grade unless otherwise specified.

### 2.2. Phase solubility studies

The phase solubility studies were carried out according to the method of Higuchi and Connors (Jansook et al. 2018; Higuchi & Connors 1965). Briefly, an excess of KME was added to 200 μL aqueous solutions of different HP-β-CD concentrations (1, 2, 4, 6, 8, and 10 mM). All samples were shaken at 25 °C and 100 rpm for 72 h until reaching equilibrium. Subsequently, the samples were withdrawn and filtered through a 0.45 µm membrane to remove undissolved KME. The concentration of KME in the HP-β-CD solutions was determined by an HPLC method, and the analyses were performed in triplicate (*n* = 3). The apparent stability constants (Ks) were calculated by the following equation (Nair et al. 2014; Pinto et al. 2020): Ks = slope/S_o_ (1-slope), where S_o_ represents the intrinsic solubility of the drug, and the slope is the slope of the linear KME-CD phase solubility diagram.

### 2.3. Preparation of KME/HP-β-CD inclusion complexes

According to the results of the phase solubility study, the required KME and HP-β-CD were weighed in a molar ratio of 1:1 to prepare KME/HP-β-CD. Briefly, KME (5 mg) was dissolved in 1 mL of methanol, and HP-β-CD (25.2 mg) was dissolved in 1 mL of distilled water. The resulting mixture solution was stirred at 50 °C for 1 h, and then the methanol was removed by rotary evaporation. The sample was centrifuged, and the supernatant was collected and freeze-drying to obtain a white powdery KME/HP-β-CD. Physical mixtures were prepared by the simple mixing of KME with HP-β-CD.

### 2.4. Complexation efficiency

The KME content in KME/HP-β-CD was determined by an HPLC method. A Shimadzu LC-20AT system (Japan) with a C_18_ column (H&E column, 150 mm × 4.6 mm, 5 μm) was used for separation. The samples were eluted in isocratic mode, and the mobile phase consisted of methanol and water (0.1% ammonia) (70:30, v/v). The flow rate was 1.0 mL/min. The detection wavelength was set at 265 nm, the injection volume was 20 μL, and the column temperature was maintained at 30 °C. The standard curve of KME (0.5–100 μg/mL) was determined to be Area_peak_ = 10735 C + 4080.7, *R*^2^ = 0.9999.

In order to determine the entrapped amount of KME in KME/HP-β-CD, 10 mg of inclusion complexes were dissolved in the mobile phase and analyzed after appropriate dilutions (*n* = 3). The complexation efficiency (CE%) was calculated by the following equation:

CE % = mass of KME in inclusion complexes/mass feeding of KME × 100%

### 2.5. Optimization of KME/HP-β-CD inclusion complexes

A 3-factor, 3-level BBD was applied to optimize KME/HP-β-CD inclusion complexes by Design-Expert version 8.0.6 software. BBD was particularly selected because it runs fewer times than the central composite design (CCD) in the case of three or four variables (Liu & Ho 2017; Khushbu 2021). A design matrix containing 17 experimental runs was established. The quadratic model generated by the nonlinear software is:

$$Y=b_{0}+b_{1}X_{1}+b_{2}X_{2}+b_{3}X_{3}+b_{12}X_{1}X_{2}+b_{13}X_{1}X_{3}+b_{23}X_{2}X_{3}+b_{11}X_{1}^{2}+b_{22}X_{2}^{2}\;+b_{33}X_{3}^{2}$$

where Y is the response function of the experimental data, X_1_, X_2_, and X_3_ are independent variables. X_1_X_2_ (X_1_X_3_, X_2_X_3_) and X_1_^2^ (X_2_^2^, X_3_^2^) represent the interaction and quadratic terms, respectively, and b_0_ is an intercept, b_1_ to b_33_ are regression coefficients (Ren et al. 2019). The dependent and independent variables and their low, medium, and high levels were shown in Table 1. The levels were selected according to the results of the single factor test (Table S1). The statistical significance of the fitted model was evaluated by analysis of variance (ANOVA). 3D response surface plots and 2 D contour plots were drawn to visualize the influence of each factor on response.

**Table 1.** Variables and observed responses in BBD design for inclusion complexes.

No.	Molar ratio* (*X_1_*)	Time (*X_2_*, h)	Temperature (*X_3_*, ^o^C)	CE (*Y*, %)
1	3	2	50	75.02
2	1	2	30	38.07
3	3	3	70	73.09
4	3	2	50	70.24
5	1	1	50	37.32
6	5	2	30	82.89
7	3	2	50	76.60
8	3	2	50	75.27
9	3	3	30	75.08
10	5	1	50	89.87
11	3	2	50	75.49
12	3	1	70	72.30
13	1	3	50	43.17
14	5	2	70	82.47
15	5	3	50	87.71
16	3	1	30	74.20
17	1	2	70	37.55

*Y* = +74.52+23.35 *X*_1_+0.67 *X*_2_−0.60 *X*_3_−2.00 *X*_1_*X*_2_+0.025 *X*_1_*X*_3_−0.022 *X*_2_*X*_3_−11.71 *X*_1_^2^+1.71 *X*_2_^2^−2.56 *X*_3_^2^; * The amount of KEM is fixed, so this variable can also be expressed as the concentration of HP-β-CD.

### 2.6. Physicochemical characterization of the optimal KME/HP-β-CD inclusion complexes

#### 2.6.1. SEM

The surface morphology of the raw KME, HP-β-CD, and KME/HP-β-CD was investigated using an SEM instrument (Quanta450, FEI, USA). The samples were mounted on aluminum stubs with double-sided carbon adhesive tape and then coated with gold. Finally, SEM was conducted at 5 kV to observe the surface morphology of the samples.

#### 2.6.2. DSC

The thermal properties of the raw KME, HP-β-CD, KME/HP-β-CD, and physical mixtures were investigated by a DSC instrument (TA Instruments, Newcastle, DE, USA). Approximately 5–10 mg of sample was sealed into an aluminum DSC pan, with the same empty pan used as a reference. The sample was scanned from 50 to 250 °C at a rate of 10 °C/min at a flow rate of 50 mL/min in an N_2_ atmosphere.

#### 2.6.3. PXRD

The PXRD patterns of the raw KME, HP-β-CD, KME/HP-β-CD, and physical mixtures were obtained by a powder X-ray diffractometer (Ultima, Shimadzu, Kyoto, Japan) employing Cu Kα radiation at 40 kV and 40 mA. All samples were evaluated at a scan rate of 2°/min over a 2θ range of 5–50°.

#### 2.6.4. FT-IR spectroscopy

The FT-IR spectra of the raw KME, HP-β-CD, KME/HP-β-CD, and physical mixtures were recorded over a spectral region of 4000 – 500 cm^−1^ by an FT-IR spectrometer (Thermo Fisher Scientific, MA, USA). All samples were prepared as KBr tablets.

#### 2.6.5. ^1^H NMR spectroscopy

The ^1^H NMR spectra of the raw KME, HP-β-CD, and KME/HP-β-CD were obtained by a Bruker NMR instrument (400 MHz; USA) to evaluate the formation of chemical bonds. Samples (3–5 mg) were dissolved in dimethyl sulfoxide-d_6_ (DMSO-*d*_6_) and then transferred to NMR tubes for ^1^H NMR data acquisition.

### 2.7. Solubility of KME and KME/HP-β-CD

Excess KME and KME/HP-β-CD were added to distilled water to obtain a supersaturated solution (Arya & Raghav 2021). The samples were shaken at 37 °C for 24 h and 100 rpm. After centrifugation, the supernatants were filtered through a 0.45 μm membrane and the solubility was determined (*n* = 3) with the validated HPLC method.

### 2.8. *In vitro* drug release study

A dialysis bag diffusion method was used to evaluate *in vitro* drug release profiles of both KME and KME/HP-β-CD (Zhao et al. 2021). 2 mg of KME was dispersed in water, and then transferred to a dialysis bag and placed in a beaker containing 40 mL of pH 6.8 phosphate buffer. The study was carried out at 37 ± 0.5 °C and a magnetic stirring speed of 100 rpm. Aliquots of approximately 4 mL were taken out at predetermined time intervals (10 min, 15 min, 30 min, 1 h, 2 h, 3 h, and 6 h) and replaced by the same volume of fresh buffer to maintain a constant volume of dissolution media during sampling. The samples were filtered and analyzed by HPLC.

### 2.9. *Ex vivo* everted gut sac experiments

The protocol of the animal study was approved by the Institutional Animal Care and Use Committee of Fujian Medical University (permit number: SYXK (Min) 2018-0001), China. Male SD rats weighing 220–250 g were fasted overnight before the experiment and with free access to water. The everted gut sac method was carried out as in previously published reports with minor modifications (Tambe et al. 2019). Rats were anesthetized and sacrificed, and the intestinal segments of interest were identified and dissected (duodenum, starting 1 cm from the stomach; jejunum, 10–18 cm from the stomach; ileum, starting 8 cm above the cecum; colon, 5 cm below the cecum) (Rong et al. 2013).

Each obtained intestinal segment was immediately washed with ice-cold Krebs-Ringer (K-R) solution, and then turned out with a glass rod, and one end was ligated with a thread. After inserting the glass tube, the other open end of the everted sacs was also tightened. Then blank K-R solution (2.5 mL) was added to the everted gut sacs. The everted segments were preincubated in 50 mL of K-R solution at 37 °C for 5 min. Then, KME, KME + 100 μg/mL verapamil or KME/HP-β-CD was added to the mucosal side of the intestinal segment to obtain a final concentration of 100 μg/mL. 1 mL of sample was taken from the serosal side at predetermined time intervals (30, 60, 90, and 120 min) and replaced by the same volume of fresh K-R solution. During the whole incubation period, a gas mixture of 95% oxygen and 5% carbon dioxide was continuously bubbled into the culture medium. After the experiment, the length of each intestinal segment was accurately measured, and the concentration of KME was measured by HPLC. The apparent permeability coefficient (Papp) values of KME were calculated by the following equation (Tambe et al. 2019):

Papp = (dQ/dt)/(A × C0), where Papp is expressed in cm/s, dQ/dt is the slope of the linear portion of the curves, A is the surface area of the gut sac, and C0 is the initial concentration of KME on the donor side (100 μg/mL).

### 2.10. Cell viability study

The cytotoxicity of KME and KME/HP-β-CD was evaluated in Caco-2 cells using the cell counting kit-8 (CCK-8) assay. Caco-2 cells were cultured in DMEM containing 20% FBS and incubated at 37 °C with 5% CO_2_. Caco-2 cells (1 × 10^4^ cell/well) were seeded in 96-well plates and incubated for 24 h. Then, 100 μL of fresh medium containing KME or KME/HP-β-CD (5–100 µg/mL) was added. After 24 h, the culture medium was changed with CCK-8 solution and the samples were incubated for another 2 h. The optical density (OD) was read at 490 nm (BioTek Instruments, Winooski).

### 2.11. Caco-2 permeability study

Caco-2 cells were seeded at a density of 5 × 10^5^ cells/well on top of 12-well Transwell® polycarbonate filters, and cultured in a 5% CO_2_ incubator at 37 °C for 21 days. The medium was changed every 2 days in the first week, and then every day for the next two weeks. At the end of 21 days, a Millicell-ERS epithelial voltmeter (Merck, USA) was used to analyze the transepithelial electrical resistance (TEER) value of Caco-2 cells. Only monolayers with TEER values higher than 600 Ω·cm^2^ can be used in subsequent transport experiments (Chen et al. 2018).

To investigate the permeability of KME and KME/HP-β-CD inclusion complexes in Caco-2 cells, the apical to basal (A-B) and basal to apical (B-A) transport of KME was measured. After preincubation for 30 min with blank HBSS, 0.5 mL of HBSS medium containing KME or KME/HP-β-CD (equivalent KME concentration of 50 μg/mL) was added to the apical side in the A-B direction experiments, and 1.5 mL of fresh HBSS was added to the basolateral side of the inserts as the acceptor phase. In contrast, for the B-A direction experiments, 1.5 mL of HBSS medium containing KME or KME/HP-β-CD (equivalent KME concentration of 50 μg/mL) was added to the basolateral side, and 0.5 mL of fresh HBSS was added to the apical side of the inserts as the acceptor phase. The plates were incubated in a shaker at 37 °C. 100 μL of the sample were taken from the acceptor side at predetermined time intervals (30, 60, 90, and 120 min) and replaced by the same volume of fresh HBSS to maintain a constant volume. Finally, the obtained samples were analyzed by HPLC. The Papp values (cm/s) were determined using the following equation (McCartney et al. 2019; Patel & Sawant 2019):

$$\mathrm{Papp}=(\mathrm{dQ}/\mathrm{dt})/(A\times C_{0}),\text{where dQ}/\text{dt is the steady}-\text{state flux},\text{ A is the surface area of the monolayer }(1.12\;{\mathrm{cm}}^{2}),\text{ and }C_{0}\text{ is the donor concentration }(50\text{ μg}/\mathrm{mL}).$$

$$\mathrm{TEER}\;(\Omega \cdot {\mathrm{cm}}^{2})=(\mathrm{TEER}\;(\Omega )\;–\text{TEER blank }(\Omega ))\;\times \;A({\mathrm{cm}}^{2})$$

Here, TEER (Ω), Caco-2 monolayers; TEER blank (Ω), the insert without cells; A (cm^2^), 1.12 cm^2^.

### 2.12. *In vivo* pharmacokinetics study

The *in vivo* pharmacokinetic study was approved by the Institutional Animal Ethical Committee, Fujian Medical University, China. Male SD rats (200 ± 20 g) have fasted for 12 h with free access to water before oral administration. The rats were divided randomly into two groups (*n* = 6) and received KME or KME/HP-β-CD by oral gavage at a drug dose of 12 mg/kg. The KME powder and KME/HP-β-CD were dispersed in deionized water containing 0.1% sodium carboxymethylcellulose (CMC-Na). Blood samples were collected at predetermined intervals (0.05, 0.1, 0.16, 0.5, 1, 3, 6, 9, and 12 h) from the retroorbital venous sinus, and placed in heparinized anticoagulant tubes. After centrifugation at 4,000 rpm for 10 min at 4 °C, the plasma was separated and stored at −80 °C until UPLC-MS/MS analysis.

Plasma samples were prepared by the protein precipitation method (Chen et al. 2013). Briefly, an aliquot of 50 μL plasma sample and 200 μL of acetonitrile containing 10 ng/mL gelsemine (internal standard, IS) were added to a centrifuge tube. The mixture was vortexed for 5 min and centrifuged at 18,000 rpm for 10 min at 4 °C. Then the supernatant was transferred and injected into the UPLC-MS/MS system for analysis. Chromatographic separation was performed using a UPLC instrument (Agilent 1290, Agilent Technologies, USA), equipped with a mass spectrometer (QTRAP^®^ 5500, AB SCIEX, USA). The mass spectrometer detector was performed with multiple reactions monitoring scan mode at m/z 307.2 → 180.1 for KME and m/z 323.1 → 236.1 for IS. More detailed information about UPLC-MS/MS is provided in the supplementary material. The main pharmacokinetic parameters such as *T*_1/2_, *C*_max_, *T*_max_, and area under the curve (AUC), were determined using the non-compartmental model with DAS 3.2.1 software. The relative bioavailability *F* was calculated by the following equation:

$$F(\%)\;=({\mathrm{AUC}}_{0–t}\;(\text{inclusion complexes})/{\mathrm{AUC}}_{0–t}(\mathrm{KME}))\;\times \;100\%$$

### 2.13. Statistical analysis

All data are presented as the means ± standard deviation (SD). Statistical analysis was conducted by Student’s *t*-test using SPSS 16.0 software. Differences were considered statistically significant at *p < .05 and **p < .01.

## 3. Results and discussion

### 3.1. Phase solubility study

**Figure 1.**
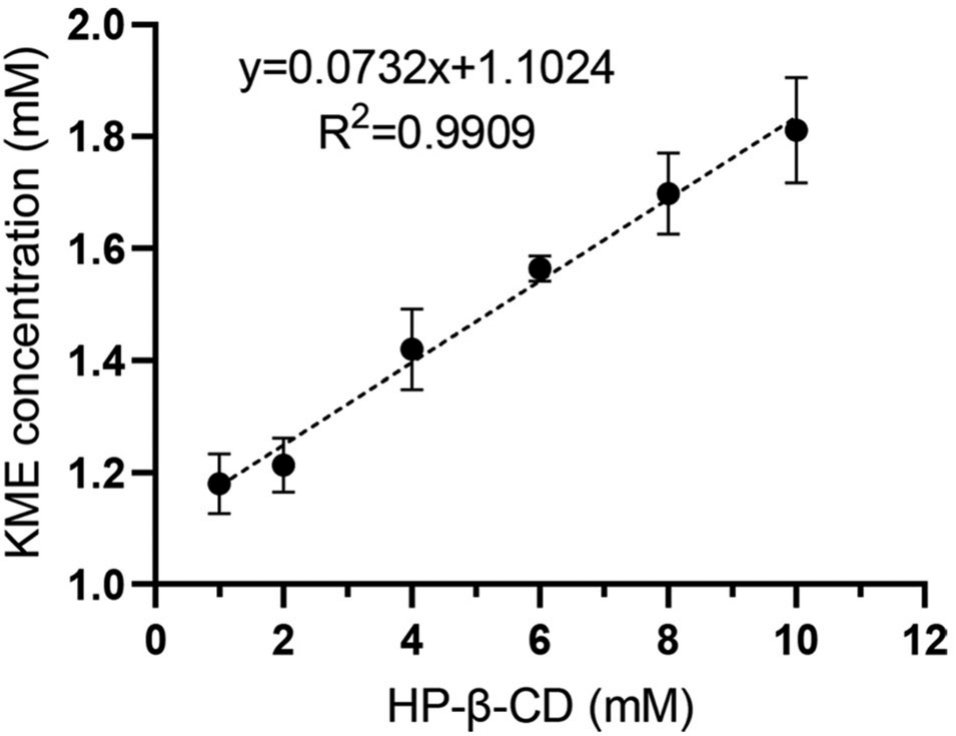
Phase solubility diagrams of the KME with different HP-β-CD concentrations in distilled water at 25 ± 0.5 °C (*n* = 3).

The phase solubility curve gives an illustrative overview of the complexation process. This result provided information on the solubility enhancement of KME after HP-β-CD complexation as well as the inclusion stoichiometry and Ks values of the complex (Pinto et al. 2020). The phase solubility curve of KME and HP-β-CD was presented in Figure 1. As the concentration of HP-β-CD increased in the range of 1–10 mM, the water solubility of KME increased linearly. The equilibrium solubility of KME in water was 0.70 ± 0.02 mg/mL, and when the concentration of HP-β-CD was 10 mM, the solubility of KME was significantly increased to 1.81 mg/mL (2.59-fold). The phase solubility curve was a typical AL-type curve, as described in solubility diagrams by Higuchi and Connors. A regression coefficient (*R*^2^) exceeding 0.99 and slope value (0.0732) of less than 1 revealed the formation of the KME/HP-β-CD system with a 1:1 stoichiometry. The Ks of KME/HP-β-CD were calculated based on the parameters of the phase solubility plot. According to the literature (Loftsson et al. 2005), optimal values for Ks range between 50 and 2000 M^−1^, a smaller value indicates that the interaction between the drug and CD is too weak, while a value higher than this range indicates that the drug is not completely released from the inclusion complexes. In our study, the Ks value was calculated to be 34.4 M^−1^, indicating a weak interaction between KME and HP-β-CD.

### 3.2. Preparation and optimization of KME/HP-β-CD inclusion complexes

The KME/HP-β-CD inclusion complexes were prepared by the solvent evaporation method. The process parameters of KME/HP-β-CD were optimized by a BBD statistical experimental design. Table 1 showed the independent variables and responses for all 17 experimental runs. Design-expert 8.0.6 software was used to fit quadratic multinomial models for the experimental values. The precision ratio of 31.9 (>4) indicated an appropriate signal-to-noise ratio. The model was statistically significant at *p* < .01 with an F-value of 118.8. The lack-of-fit *F*-value was 0.46, which implied that the lack of fit was nonsignificant and hence the model was valid for further study (Table S2). The model described could be represented as follows:

$$Y=+74.52+23.35\;X_{1}+0.67\;X_{2}-0.60\;X_{3}-2.00\;X_{1}X_{2}+0.025\;X_{1}X_{3}-0.022\;X_{2}X_{3}-11.71\;X_{1}^{2}+1.71X_{2}^{2}-2.56X_{3}^{2}$$

**Table 2.** Variation of ^1^H-NMR chemical shifts (δ, ppm) data of HP-β-CD and KME in inclusion complexes.

HP-β-CD	δ _HP-β-CD_	δ _KME/HP-β-CD_	Δδ (ppm)
H1	5.0308	5.0304	−0.0004
H2	3.6151	3.6146	−0.0004
H3	3.7534	3.7508	−0.0025
H4	overlap with DMSO-*d*6		
H5	3.5684	3.5697	−0.0013
H6	3.5843	3.5841	−0.0002
KME	δ_KME_	δ _KME/HP-β-CD_	Δδ（ppm）
H12	7.5812	7.5853	0.0041
H9	7.5173	7.5202	0.0029
H11	7.3406	7.3442	0.0036
H10	7.2536	7.2576	0.0040

**Figure 2.**
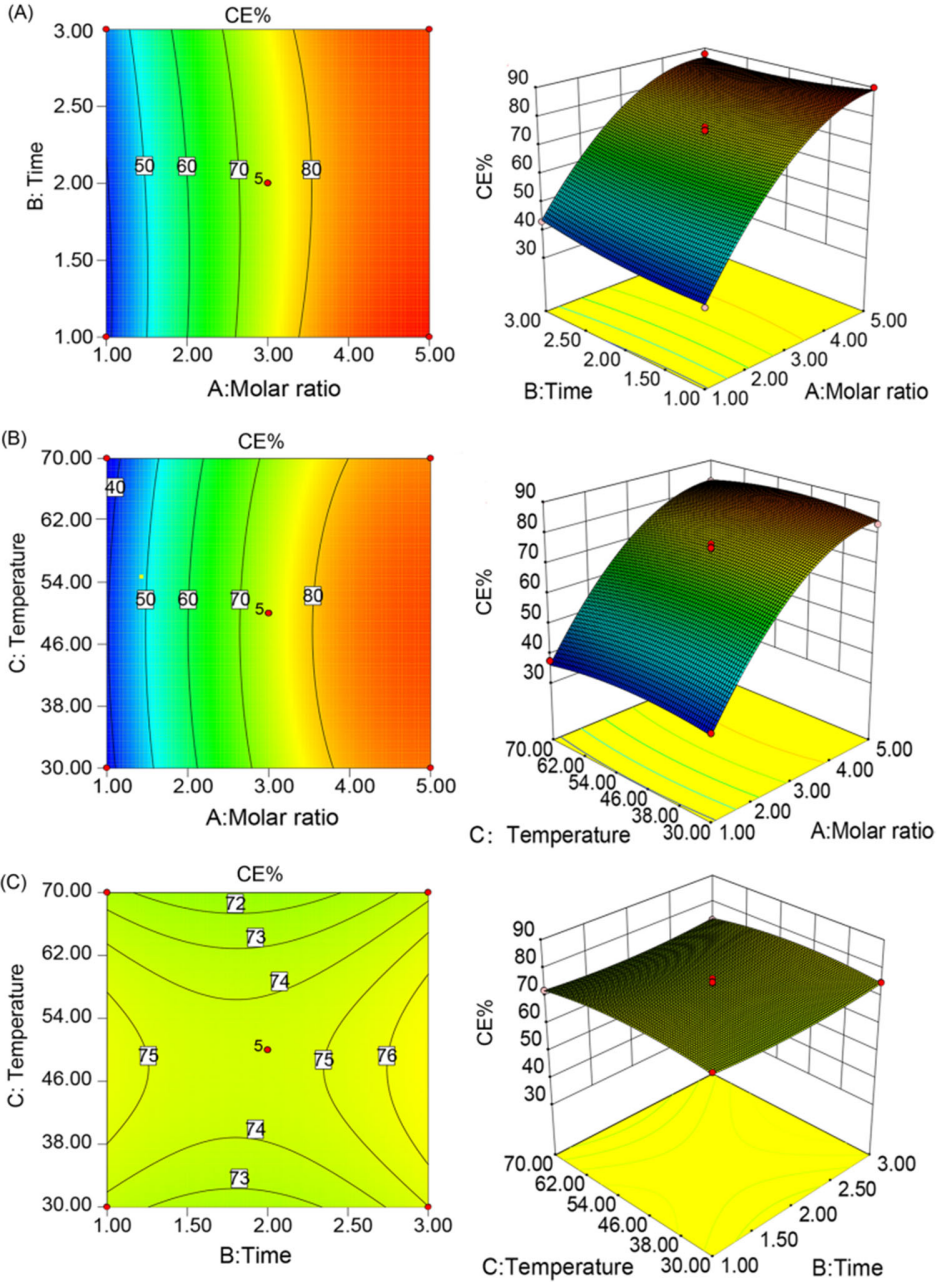
Contour plot and 3 D-response surface plot for the effects of temperature, time, and the molar ratio of HP-β-CD with KME on the CE% of inclusion complexes.

ANOVA revealed the significant positive effect of the molar ratio of HP-β-CD with KME on the CE% (*p* < .0001), which was also proved by the positive sign of the coefficients X_1_. 3 D response surface plots and contour plots were used to illustrate the interactions between formulation variables and the dependent variable. These types of plots help to study the effects of two factors on the response at the same time (Ren et al. 2019). In all the figures presented, the third factor remained at a constant level. As shown in Figure 2, X1 has a positive effect on CE%, while variation in X_2_ and X_3_ has no significant effect on CE%. The CE% values ranged from 37.32% to 89.87%. The optimal process parameters were determined by applying the point prediction methods in Design-Expert Software. The optimal parameters with a maximized CE% were a molar ratio of KME to HP-β-CD of 1:5, a stirring temperature of 50 °C and an inclusion time of 1 h. The optimal formulation was prepared, and the actual values (89.90 ± 0.28% for CE%) were close to the predicted value (89.81% for CE%). The relative deviation (%) was below 5%, indicating that the BBD statistical experimental design can accurately predict the CE% of the inclusion complexes.

### 3.3. Physicochemical characterization of the optimal KME/HP-β-CD inclusion complexes

#### 3.3.1. SEM

**Figure 3.**
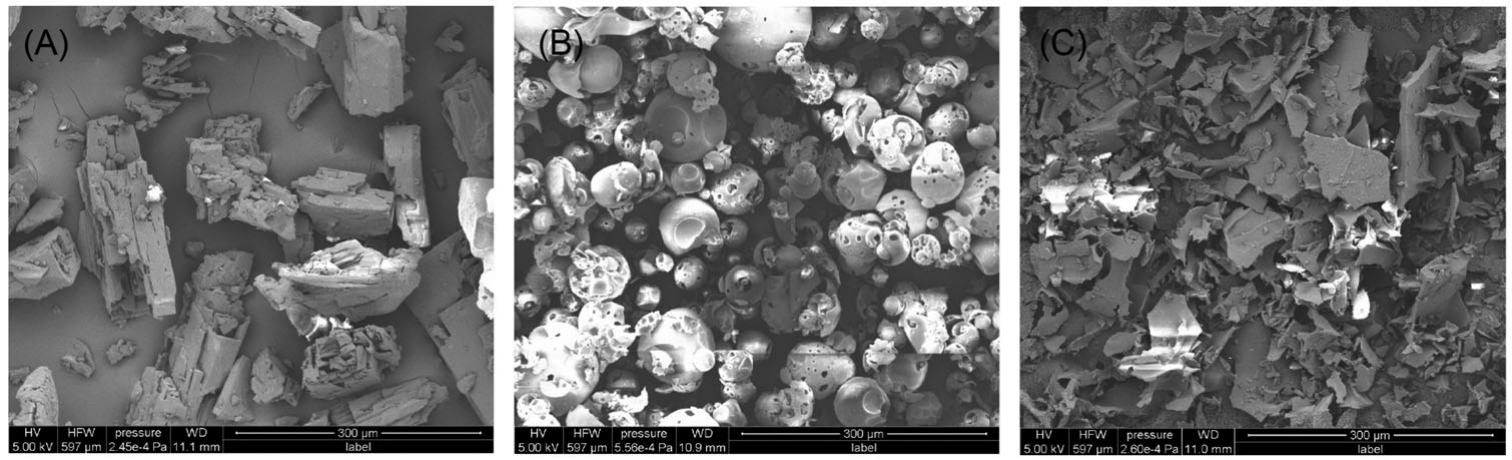
SEM images of (A) raw KME, (B) HP-β-CD, and (C) KME/HP-β-CD inclusion complexes.

SEM images of the raw KME, HP-β-CD, and KME/HP-β-CD were presented in Figure 3. SEM micrographs of the raw KME showed prismatic crystals and compact structures (Figure 3(A)), and HP-β-CD showed amorphous spherical particles (Figure 3(B)). The prepared KME/HP-β-CD exhibited an amorphous sheet structure (Figure 3(C)), which was different from that of either KME or HP-β-CD. It was difficult to distinguish KME from HP-β-CD in the inclusion complexes. These observations revealed that crystalline KME might have been complexed in the cavity of HP-β-CD, indicating the formation of a new solid phase complex. The solid-state properties of the samples were further determined by DSC and PXRD.

#### 3.3.2. DSC

**Figure 4.**
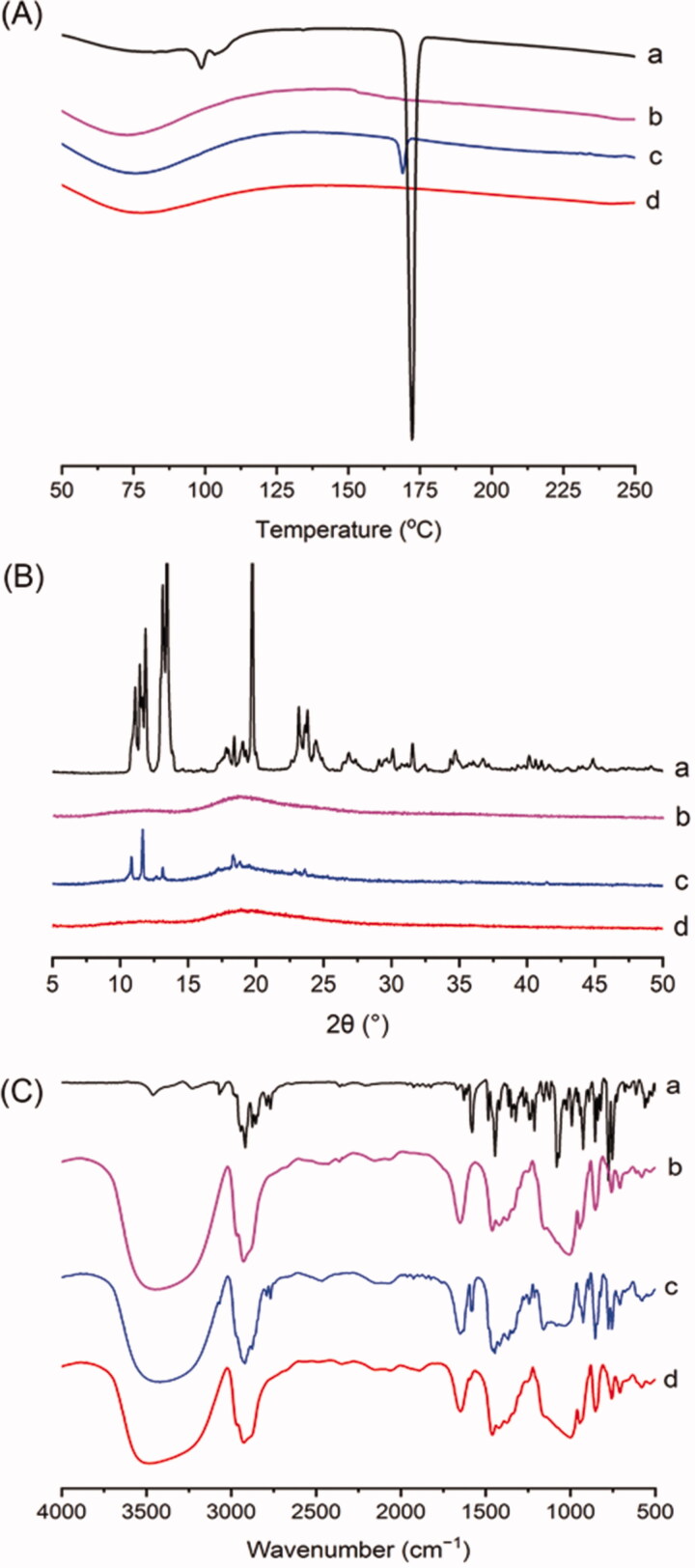
Physicochemical characterization: (A) DSC curves, (B) PXRD patterns, and (C) FT-IR spectra of (a) KME; (b) HP-β-CD; (c) physical mixture; and (d) KME/HP-β-CD inclusion complexes.

DSC thermograms of the raw KME, HP-β-CD, physical mixtures, and KME/HP-β-CD were presented in Figure 4(A). The DSC thermogram of raw KME showed a characteristic sharp endothermic peak at 172 °C, which indicates its crystalline state (Figure 4(A-a)). HP-β-CD did not demonstrate any crystallinity (Figure 4(A-b)). Moreover, the physical mixtures showed a distinct endothermic peak from KME but with reduced intensity and a slight shift from its initial position (Figure 4(A-c)). These results suggested that KME was present in a crystalline state in the physical mixtures. However, the endothermic peak completely disappeared in the KME/HP-β-CD thermogram (Figure 4(A-d)), indicating the formation of inclusion complexes. Additionally, the KME molecules might have become embedded into the CD cavity, resulting in the transformation from the crystalline drug to its amorphous nature. This result was further supported by the PXRD study.

#### 3.3.3. PXRD

Polymorphic changes in an active drug are important because such a transition may affect the solubility and bioavailability of the drug (Khalid et al. 2018). The PXRD results of the raw KME, HP-β-CD, physical mixtures, and KME/HP-β-CD were shown in Figure 4(B). Raw KME exhibited a series of high-intensity peaks at 2θ values of approximately 11.09°, 11.44°, 11.86°, 12.97°, 13.46°, and 19.48° (Figure 4(B-a)), indicating its highly crystalline nature. The PXRD pattern of HP-β-CD has no crystal peak, which implies that HP-β-CD is amorphous in nature (Figure 4(B-b)). The peaks corresponding to KME with reduced intensity could still be observed in the PXRD pattern of the physical mixtures (Figure 4(B-c)), suggesting that KME still existed in its crystalline form. One broad hallow was found in the spectrum of KME/HP-β-CD (Figure 4(B-d)), which was not the characteristic peak of raw KME, but an amorphous halo pattern similar to the spectrum of HP-β-CD. The results further confirmed the formation of KME/HP-β-CD in an amorphous state.

#### 3.3.4. FT-IR

FT-IR is widely used to analyze the interactions between guest molecules and CD in the solid-state (Braga et al. 2021). FT-IR spectra of the raw KME, HP-β-CD, physical mixtures, and KME/HP-β-CD were shown in Figure 4(C). The peaks of KME were found at 2925 cm^−1^ (C–H stretching), 1578 cm^−1^ (C=N stretching), 1450 cm^−1^ (benzene skeleton vibration), and 1080 cm^−1^ (C–O–C bond stretching vibrations) (Figure 4(C-a)). The broad absorption band at 3427 cm^−1^ shown in the HP-β-CD was attributed to the stretching vibration of the free OH group. Other prominent absorption bands of C–H, H–O–H bending, C–O, and C–O–C stretching vibrations in HP-β-CD were observed at 2925 cm^−1^, 1650 cm^−1^, 1151 cm^−1^, and 1012 cm^−1^, respectively (Figure 4(C-b)). Similar characteristic absorption bands from KME and HP-β-CD were observed in the spectrum of the physical mixtures (Figure 4(C-c)), demonstrating a weak interaction between KME and HP-β-CD. However, the characteristic C=N stretching vibration peak from KME at 1578 cm^−1^ disappeared in KME/HP-β-CD (Figure 4(C-d)), indicating possible encapsulation of the phenylindole moiety into the cavity of HP-β-CD. This change was attributed to the inclusion of HP-β-CD, which destroyed the coplanar properties of conjugated rings and leaded to the disappearance of absorption peaks (Zhang et al. 2009).

#### 3.3.5. ^1^H NMR

**Figure 5.**
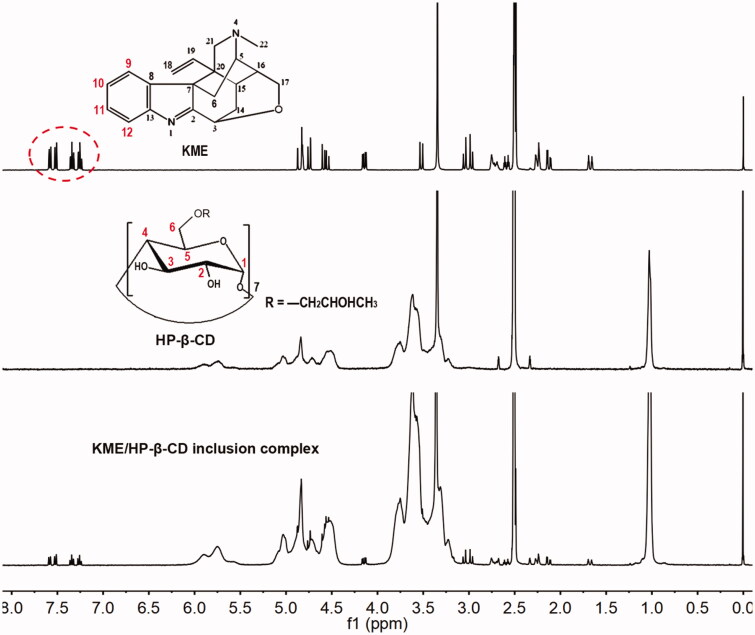
Physicochemical characterization: ^1^H NMR spectrum of KME, HP-β-CD, and KME/HP-β-CD inclusion complexes.

NMR spectroscopy provides important information about the interaction between guest molecules and host molecules. The entry of drug molecules into the HP-β-CD cavity can be analyzed by observing the changes in the chemical shifts (δ) of the protons in the complex. The ^1^H NMR spectra of KME, HP-β-CD, and KME/HP-β-CD in DMSO*-d_6_* were presented in Figure 5. In the presence of KME, the δ of HP-β-CD were changed due to the formation of the inclusion complexes. Table 2 showed a comparison of the δ of HP-β-CD protons with and without KME. The chemical shift variations (Δ) were calculated by the equation Δ = δ (complex) – δ (free). As KME was added into the CD cavity, the H-3 and H-5 protons in the ^1^H NMR spectrum of HP-β-CD exhibited an upfield shift, pointing to the interior of the cavity and indicating the formation of an inclusion complex. However, the interaction between KME and cyclodextrin molecules can be observed to be weak because the chemical shift variations are slight. Additionally, It has been reported that when ΔH3 > ΔH5, part of the drug is included in the cavity, and when ΔH3 ≤ΔH5, total inclusion takes place (Silva et al. 2021). In our study, KME/HP-β-CD presented ΔH3 > ΔH5, indicating that part of KME was contained in the cavity of the CD molecule, resulting in less stable inclusion complexes. The result was in accordance with the phase solubility study.

By comparing the ^1^H NMR spectrum of KME with and without HP-β-CD, the inclusion mode of KME/HP-β-CD was further evaluated. After complexation with HP-β-CD, changes in the downfield chemical shifts between 7.0 and 8.0 ppm (Δ 0.0029–0.0041) were observed, as also shown in Table 2, indicating that the aromatic protons of KME are involved in inclusion complexes. The result was in accordance with the FT-IR analysis, and the phenylindole moiety of KME may be embedded in the hydrophobic cavity of HP-β-CD. A similar change in the δ due to the complexation of the drug molecule with the CD moiety has been reported in prior studies (Giri et al. 2021). Of course, further investigations, including molecular docking studies, might be important to understand these experimental data.

### 3.4. Solubility and in vitro release study

The water solubility of the KME or KME/HP-β-CD was evaluated by preparing saturated solutions. The solubility of KME was found to be poor at 0.70 mg/mL, but increased significantly to 36.64 mg/mL after complexation with HP-β-CD; Compared with free KME, the solubility of KME/HP-β-CD was increased by 52.34 times. The result indicated that the solubility of KME can be successfully modified by inclusion complexes, which will help improve the bioavailability of the drug.

**Figure 6.**
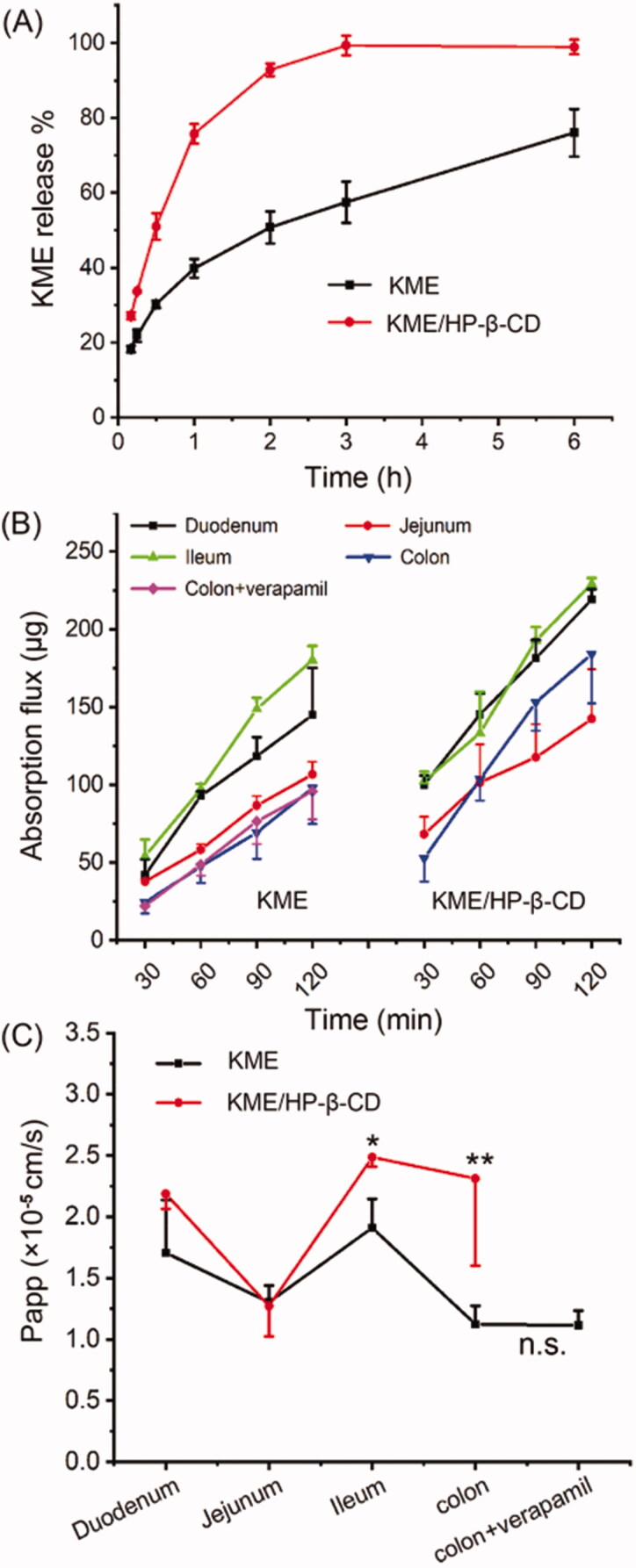
(A) *In vitro* release profiles of KME and KME/HP-β-CD using dialysis bag technique in PBS buffer (pH 6.8). (B) The regional intestinal absorption of KME and KME/HP-β-CD in everted gut sac model. (C) The Papp values of KME and KME/HP-β-CD across the different intestinal regions. Data are shown as mean ± SD (*n* = 3). *p < .05, **p < .01, compared to the same segment of KME group; n.s., no significant difference, colon compared to the colon + verapamil (100 μg/mL).

*In vitro* release studies are very important during the development of a dosage form and ultimately for quality control. The release profiles of KME and KME/HP-β-CD in pH 6.8 buffer were shown in Figure 6(A). KME was used as a control group, and its release rate was determined to be 40% in the first 1 h and reached 70% within 6 h. KME/HP-β-CD showed a faster dissolution rate than KME. The release rate was close to 90% within 3 h and then reached a plateau. To gain insight into the kinetics of KME release from the inclusion complexes, the obtained data were fitted to various mathematical models. The curve fitting results were listed in Table S3 and were compared based on the determination coefficient value. The KME followed the Ritger-Peppas model (*r* > 0.99), and KME/HP-β-CD followed the first-order model (*r* > 0.99) over a period of 6 h.

**Table 3.** The main pharmacokinetic parameters after oral administration of the raw KME and KME/HP-β-CD inclusion complexes in rats (mean ± SD, *n* = 6).

Parameters	Raw KME	KME/HP-β-CD
*C*_max_ (ng/mL)	22.83 ± 15.62	49.93 ± 15.09*
*T*_max_ (h)	0.33 ± 0.20	0.30 ± 0.23
*T*_1/2_ (h)	0.86 ± 0.12	1.34 ± 1.03
AUC_0–12_ (ng/mL·h)	26.48 ± 17.80	72.50 ± 15.25**
AUC_0-∞_ (ng/mL·h)	30.20 ± 16.60	77.87 ± 15.18**
*F* (%)	273.79	

*p < .05; **p < .01, compared to raw KME.

### 3.5. *Ex vivo* everted gut sac experiments

*Ex vivo* everted gut sac is a very useful tool for assessing the rate, extent, and mechanism of intestinal drug absorption (Rong et al. 2013). It is a simple, fast, economic, and reproducible method for predicting drug permeability and evaluating the performance of drug delivery systems (Wang et al. 2018). The *ex vivo* rat permeation profile and intestinal apparent permeability results were presented in Figure 6(B) and Figure 6(C). As shown in Figure 6(B), both KME and KME/HP-β-CD could be absorbed into the intestinal sac. With increasing time, the absorption flux increases linearly and does not reach saturation within 120 min. The absorption of KME/HP-β-CD was better than that of KME.

The *ex vivo* permeability of KME and KME/HP-β-CD exhibited regional differences in the everted gut sac. The absorption flux of KME in each intestinal segment followed the order ileum > duodenum > jejunum > colon. The flux of KME in the colon was the smallest. According to reports, the expression level of P-gp is highest in the colon (Ho et al. 2003; Chen et al. 2018). P-gp mediated efflux might affect the absorption of KME in the colon. So, the time-dependent absorption of KME was also evaluated in the absence and presence of verapamil (100 μg/mL, inhibitor of P-gp). When verapamil was added to the colon segment, no significant difference was observed in KME absorption. The result showed that P-gp might have no significant effect on the intestinal absorption of KME in rats.

The absorption flux of KME/HP-β-CD in the duodenum, ileum, and colon was significantly higher than that in the KME group (*p < .05; **p < .01; **p < .05, respectively). Compared with the KME group, the Papp value of KME/HP-β-CD was also significantly increased in the ileum and colon (Figure 6(C), *p < .05; **p < .01, respectively). The enhanced absorption of KME in KME/HP-β-CD could be attributed to improved wettability, decreased KM crystallinity, and solubilizing effects of HP-β-CD. These properties increase the solubility of KM and create a relatively high concentration gradient across the intestinal barrier. Additionally, it has been reported that the optimum concentration of CD can enhance permeability across the intestine or any other biological barrier (Vikas et al. 2018).

### 3.6. Cytotoxicity and Caco-2 permeability study

**Figure 7.**
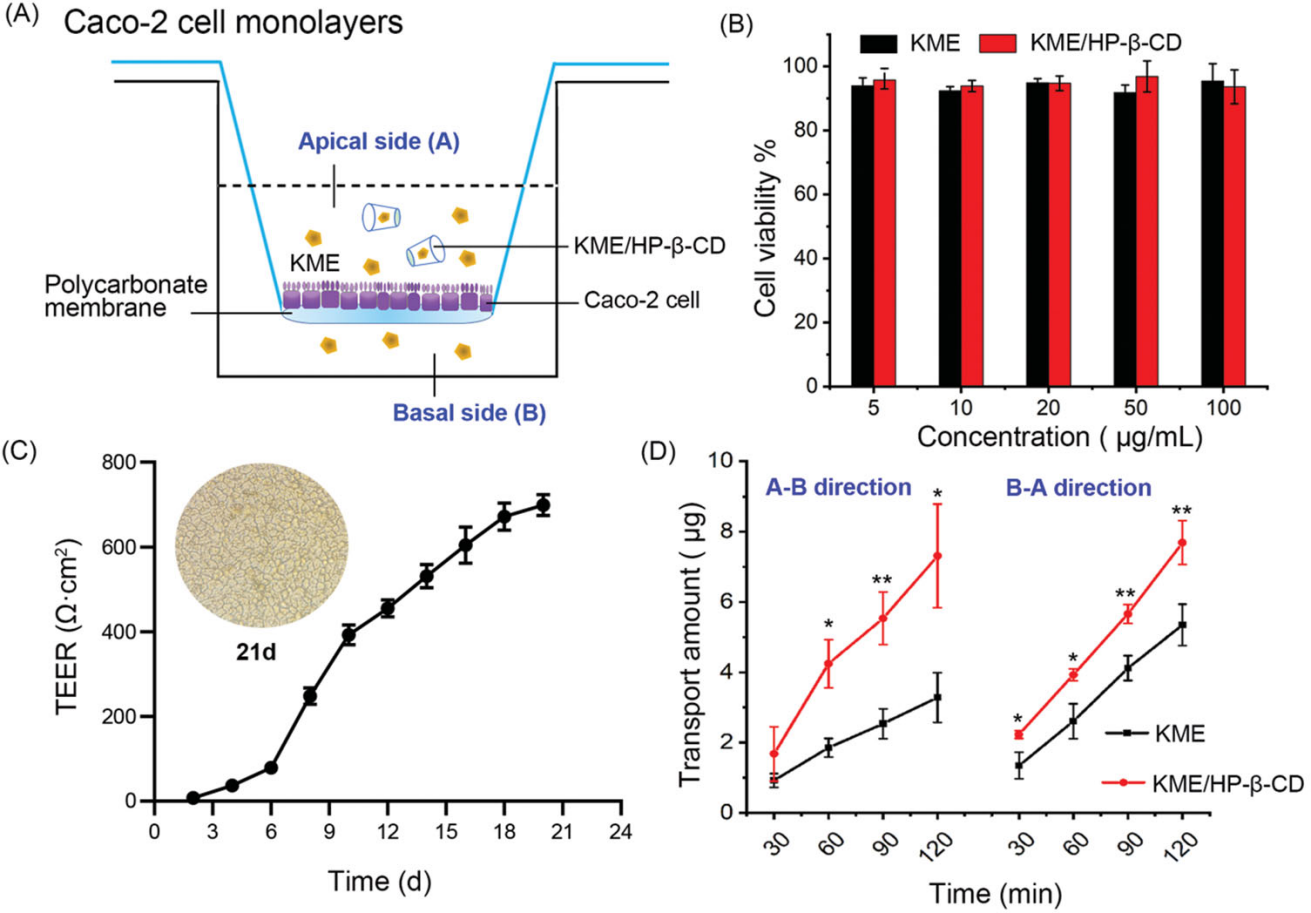
Caco-2 permeability study. (A) Schematic diagram of Caco-2 cell monolayers. (B) Cytotoxicity of KME and KME/HP-β-CD inclusion complexes in Caco-2 cells. Cells were incubated with KME or KME/HP-β-CD at 5–100 μg/mL for 24 h and evaluated by CCK-8 assay. Values are means ± SD (*n* = 6). (C) Transepithelial electrical resistance (TEER) of Caco-2 cell monolayers was measured by a Millicell-ERS epithelial voltmeter from day 0 to day 21. (D) The transport amount of KME and KME/HP-β-CD across Caco-2 cells monolayers in 120 min. Data are shown as mean ± SD (*n* = 3). *p < .05, **p < .01, compared to the KME group.

Schematic diagram of Caco-2 cell monolayers was shown in Figure 7(A). The viability of Caco-2 cells after treatment with KME was greater than 90% (Figure 7(B)), indicating that KME was nontoxic and biocompatible in the concentration range of 5–100 μg/mL. Similarly, the cell viability of Caco-2 cells treated with KME/HP-β-CD was also over 90%, suggesting HP-β-CD at a concentration of 100 μg/mL was a safe formulation vehicle for KME. Thus, an intermediate concentration of 50 μg/mL KME was noncytotoxic and suitable for permeability studies.

The Caco-2 cell monolayer experiment is regarded as a favorable tool to screen the transport efficiency of new formulations in pharmaceutical studies. Therefore, this model was established to study the transport characteristics of KME and KME/HP-β-CD. The TEER value of the Caco-2 cell monolayers was measured as an indicator of the integrity of the epithelial barrier. As shown in Figure 7(C), the cell resistance slowly increased during the first 6 days. The TEER value then increased exponentially from the 6th day to the 10th day, reaching 600 Ω·cm^2^ on the 16th day, and continued to increase. Figure 7(C) also showed a photograph of the Caco-2 cell monolayer on the 21st day, where the Caco-2 cells were observed to fuse into a continuous and intact monolayer of cells.

The flux of KME and KME/HP-β-CD across the Caco-2 cell monolayers in the A-B and B-A directions was presented in Figure 7(D). Figure 7(D) showed that the flux increased in a time-dependent manner. Over time, the transport showed an upward trend, and both directions had the same absorption characteristics. In general, the permeability coefficient for complete absorption of the drug is >1 × 10^−6 ^cm/s (Lo 2003). The Papp _A-B_ and Papp _B-A_ values for KME were (7.78 ± 2.01) ×10^−6 ^cm/s and (13.54 ± 1.72) × 10^−6 ^cm/s, respectively, indicating that KME is a highly permeable drug. Moreover, according to reports (Nagayasu et al. 2019), when the efflux ratio (B-A permeability/A-B permeability) is greater than 2, drug efflux occurs. The efflux ratio of KME was calculated to be 1.74, which suggested that KME was not an efflux pump substrate. The Papp _A-B_ and Papp _B-A_ values for KME/HP-β-CD inclusion complexes was (18.38 ± 6.24) × 10^−6 ^cm/s and (18.28 ± 2.19) × 10^−6^ cm/s, respectively. KME/HP-β-CD showed a higher Papp value (*p < .05) and lower efflux ratio (1.00) than KME itself. The result indicated that KME/HP-β-CD enhanced the absorptive permeability of KME. Thus, both the *ex vivo* everted gut sac and the permeability studies in Caco-2 cells jointly confirmed the enhanced transport efficiency of KME/HP-β-CD, which will be further verified by pharmacokinetic studies in rats.

### 3.7. *In vivo* pharmacokinetic study

**Figure 8.**
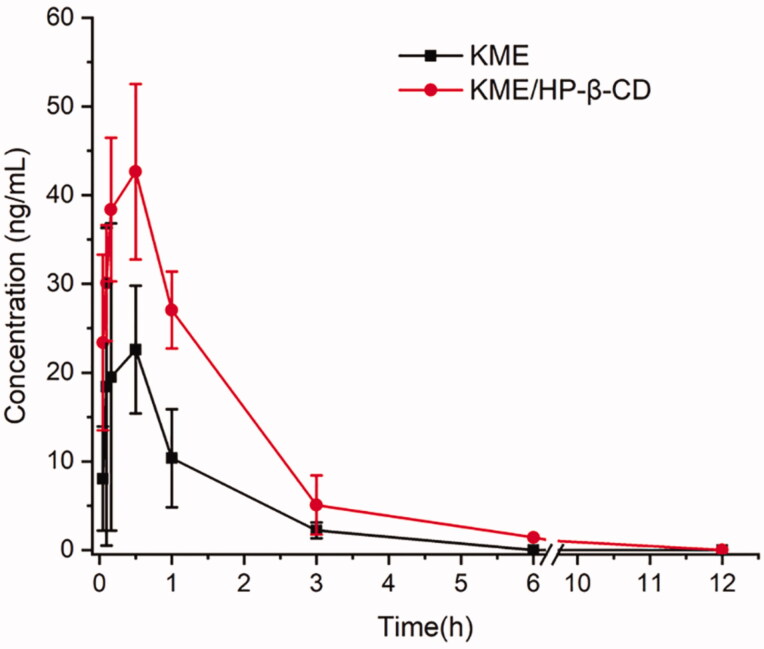
Mean plasma concentration-time curve following oral administration of the KME and KME/HP-β-CD inclusion complexes in rats (mean ± SD, *n* = 6).

According to the experimental parameters from published reports, it is found that the absolute bioavailability (Fabs) of KME is only 1% (Wang et al. 2019). However, the pharmacokinetics of KME in rats and beagle dogs have also been previously investigated in our laboratory. The Fabs of KME in the rats and beagle dogs were 14.30% and 5.67%, respectively. The oral bioavailability of KME may be relatively low, indicating its poor solubility and extensive metabolism. The adopted analytical method has been verified in terms of selectivity, linearity, and the lower limit of quantification, precision and accuracy, extraction recovery and matrix effect, stability. The results were shown in Supplementary materials. The mean plasma KME concentration-time profiles following oral administration of the KME and KME/HP-β-CD in rats were presented in Figure 8, and the main pharmacokinetic parameters were listed in Table 3. At almost all time points, the plasma concentration of KME from KME/HP-β-CD was higher than that of raw KME, which indicated that the CD formulation could effectively enhance the plasma level of KME. Compared with the KME suspension, KME/HP-β-CD showed great enhancement in the *C*_max_ and AUC_0-t_ values after oral administration. Statistically, the *C*_max_ of KME from KME/HP-β-CD was 49.93 ± 15.09 ng/mL, which was a significant (2.2-fold) improvement compared with the KME suspension (22.83 ± 15.62 ng/mL) (*p < .05). In particular, the AUC_0-t_ of KME from KME/HP-β-CD complex (26.48 ± 17.80 ng/mL·h) increased by 2.74-fold compared with that of the KME suspension (72.50 ± 15.25 ng/mL·h) (**p < .01), indicating that the oral bioavailability of KME increased by 2.74-fold through complexation with HP-β-CD. The incremental bioavailability of KME may be attributed to the increased solubility of KME when included in the inclusion complexes, allowing the drug to be kept its soluble form in the gastrointestinal tract. Moreover, the observed enhancement in intestinal permeability may also contribute to the enhanced bioavailability of KME, suggesting that the inclusion complexes are a preferable strategy to improve the properties of insoluble alkaloid components in medicinal plants.

## 4. Conclusion

KME is an active alkaloid with poor solubility and low bioavailability. In this paper, we demonstrated the feasibility of improving the oral bioavailability of KME by formulating inclusion complexes with HP-β-CD. The process parameters of KME/HP-β-CD optimized by BBD were a molar ratio of 1:5, the stirring temperature of 50 °C, and the inclusion time of 1 h. SEM, DSC, PXRD, FT-IR, and ^1^H NMR data indicated the formation of KME/HP-β-CD inclusion complexes. In addition, KME/HP-β-CD showed greatly increased water solubility, a higher release rate, and enhanced permeation when compared with KME. Furthermore, the significant increase in the AUC value of the inclusion complexes after oral administration indicated an increase in the rate and extent of KME absorption. Based on these results, we are convinced that HP-β-CD complexation is a simple and effective strategy to enhance the biopharmaceutical properties of KME, which is poorly soluble in water.

## Supplementary Material

Supplemental Material
