# Supplementary material for: Enhanced oral bioavailability of koumine by complexation with hydroxypropyl-β-cyclodextrin: preparation, optimization, ex vivo and in vivo characterization
Source: Drug Deliv. 2021 Nov 12;28(1):2415–26. doi: 10.1080/10717544.2021.1998248 (PMC8592623; doi:10.1080/10717544.2021.1998248)
Supplement: Supplemental Material [file IDRD_A_1998248_SM7296.docx]

**Supplementary materials**

**1. Single factor test**

Table S1-1 Effect of molar ratio of KME with HP-β-CD on the CE% (Mean ± SD, n=3)

| Molar ratio | CE% |
| --- | --- |
| 1:1 | 35.64±2.76 |
| 1:3 | 79.19±0.21 |
| 1:5 | 89.80±2.42 |
| 1:10 | 83.26±1.06 |

Temperature: 50 ^o^C; Time: 1 h

Table S1-2 Effect of temperature on the CE% (Mean ± SD, n=3)

| Temperature (^o^C) | CE% |
| --- | --- |
| 30 | 71.62±1.25 |
| 50 | 79.19±0.21 |
| 70 | 76.42±0.42 |

molar ratio: 1:3; Time: 1 h

Table S1-3 Effect of time on the CE% (Mean ± SD, n=3)

| Time (h) | CE% |
| --- | --- |
| 1 | 89.80±2.42 |
| 2 | 90.44±1.30 |
| 3 | 85.07±1.99 |
| 6 | 72.27±1.37 |

Temperature: 50 ^o^C; molar ratio: 1:5

Table S2 ANOVA analysis of variance for quadratic model of the CE%

|  | Sum of |  | Mean | F | p-value |
| --- | --- | --- | --- | --- | --- |
| Source | Squares | df | Square | Value | Prob > F |
| Model | 5009.62 | 9 | 556.62 | 118.84 | < 0.0001 |
| *X_1_* | 4363.05 | 1 | 4363.05 | 931.49 | < 0.0001 |
| *X_2_* | 3.60 | 1 | 3.60 | 0.77 | 0.4100 |
| *X_3_* | 2.92 | 1 | 2.92 | 0.62 | 0.4560 |
| *X_1_X_2_* | 16.05 | 1 | 16.05 | 3.43 | 0.1066 |
| *X_1_X_3_* | 0.0025 | 1 | 0.0025 | 0.000534 | 0.9822 |
| *X_2_X_3_* | 0.002025 | 1 | 0.002025 | 0.000432 | 0.9840 |
| *X_1_^2^* | 577.73 | 1 | 577.73 | 123.34 | < 0.0001 |
| *X_2_^2^* | 12.29 | 1 | 12.29 | 2.62 | 0.1492 |
| *X_3_^2^* | 27.69 | 1 | 27.69 | 5.91 | 0.0453 |
| Residual | 32.79 | 7 | 4.68 |  |  |
| Lack of Fit | 8.35 | 3 | 2.78 | 0.46 | 0.7279 |
| Pure Error | 24.44 | 4 | 6.11 |  |  |
| Cor Total | 5042.41 | 16 |  |  |  |

Table S3 Release curve fitting results of the mathematical models

| Models | KME | | KME/HP-β-CD | |
| --- | --- | --- | --- | --- |
|  | Fitting equation | r | Fitting equation | r |
| Zero-order | *Q* = 9.44+24.63 t | 0.913 | *Q* = 11.63+46.90 t | 0.623 |
| First-order | ln *Q* = - t+4.20 | 0.881 | ln *Q* = -1.5 t+4.59 | 0.993 |
| Higuchi | *Q* =27.94t ^0.5^+9.28 | 0.991 | *Q* = 37.73t ^0.5^+24.09 | 0.813 |
| Ritger-Peppas | *Q* = 38.45(t ^0.38^) | 0.997 | *Q* = 65.11(t ^0.31^) | 0.876 |

**Mass conditions and chromatographic conditions**

KME concentration in plasma was analyzed using an UPLC instrument (Agilent 1290, Agilent Technologies, USA) equipped with a mass spectrometer (QTRAP® 5500, AB SCIEX, USA). All samples were detected by a reversed phase C_18_ column (ZORBAX XDB, 4.6 mm × 50 mm, 3.5 μm Agilent). The mobile phase was composed of methanol and water with 0.1% (v/v) formic acid, and followed a gradient elution (0 - 1 min, 20% methanol; 4 - 5 min, 95% methanol; 5-10 min, 20% methanol). The column temperature was maintained at 40^o^C and the flow rate was set to 0.4 mL/min. The injection volume was 5 μL. Multiple reaction monitoring (MRM) mode was optimized as scan type. The polarity was set as positive mode. The parameters of fragmentor and collision were sat as 62.08 V and 34.01 V, respectively. And m/z =50-1000. Uncluster voltage (DP): 197.28 V (KME), 184.83 V (GM); MRM ion pair: 307.2→180.1 (KME), 323.1→236.1 (GM); Residence time: 500 ms.

**Method validation**

*Selectivity*: As shown in Figure S1, no endogenous interference was observed at the retention times of KME (3.50 min) and IS (3.88 min) in blank plasma, spiked plasma, and plasma 30 min after oral administration of KME.

*Linearity and the lower limit of quantification (LLOQ)*: The fitted linear regression equation for the calibration curve in the range of 1.0-500 ng/mL was *A* = 0.0133*C* + 0.00697, R^2^ = 0.9986, where *A* is the peak area ratio of KME and IS, and *C* is the KME concentration (ng/mL). The LLOQ of existing UPLC-MS/MS method was 1 ng/mL and a relative error (RE) accuracy of 9.0% (Table S4).

*Precision and accuracy*: The precision (as RSD%) and accuracy (as RE%) were determined by analysis of QC samples with six replicates on three consecutive days. As shown in Table S5, the RSD% of intra-and inter-day precision was < 10%, and the accuracy ranged from -4.8 to 9.6%, all within the acceptable criteria.

*Extraction recovery and matrix effect*: The recovery of KME in plasma at each QC level ranged from 86.3-93.7% with an RSD of less than 7.4%, and the matrix effect varied from 77.2-82.5% with an RSD of less than 6.9% (Table S6). No significant matrix effect for KME was observed, indicating ion suppression or enhancement from plasma was negligible in this study.

*Stability*: Table S7 showed that QC samples were stable at room temperature for 6 h, frozen -20 °C and through three freeze-thaw cycles, and post-preparation storage for 24 h.


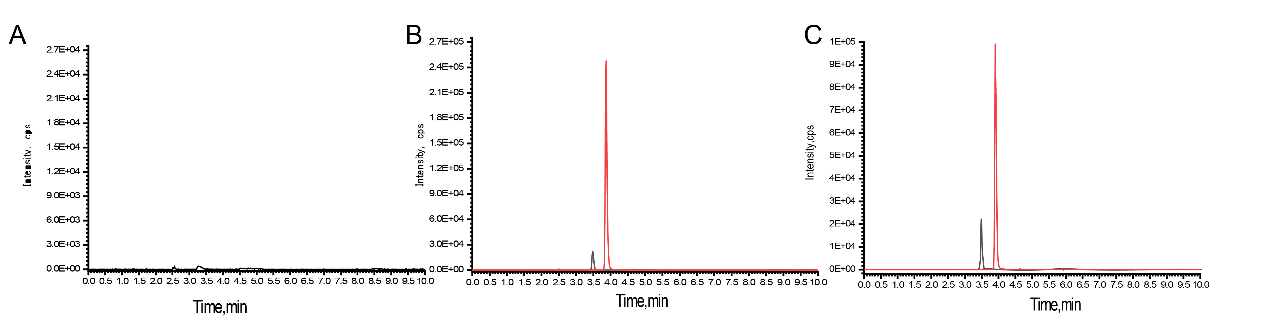


Figure 1 Representative MRM chromatograms of (A) blank plasma; (B) blank plasma spiked with KME (3.50 min) and IS (3.88 min); (C) plasma collected at 30 min after oral administration of 12 mg/kg KME.

Table S4 LLOQ of KME in plasma samples of rat (mean ± SD，n=6)

| Spiked  concentration (ng/mL) | Measured  concentration (ng/mL) | Accuracy (%) | RSD (%) |
| --- | --- | --- | --- |
| 1 | 1.09 ±0.11 | 9% | 10.0 |

Table S5 Precision and accuracy of the determination of the KME in rat plasma (n = 6).

| Spiked  Concentration  (ng/mL) | | Measured  concentration (ng/mL) | Accuracy,  RE (%) | Intra-Day  RSD (%) | Inter-Day RSD (%) |
| --- | --- | --- | --- | --- | --- |
| 1 | 0.93 ± 0.09 | | -7.0 | 1.94 | 5.10 |
| 10 | 9.52 ± 0.23 | | -4.8 | 2.42 | 7.67 |
| 100 | 94.16±1.59 | | -5.8 | 1.69 | 3.14 |
| 400 | 361.60± 6.43 | | -9.6 | 1.78 | 2.18 |

Table S6 Matrix effects and extraction recovery for the KME in rat plasma (n=6).

| Spiked  Concentration  (ng/mL) | Recovery | | Matrix effects | |
| --- | --- | --- | --- | --- |
|  | Mean% | RSD (%) | Mean (%) | RSD (%) |
| 1 | 86.3 | 7.4 | 82.5 | 6.9 |
| 100 | 87.1 | 4.0 | 79.6 | 4.6 |
| 400 | 93.7 | 3.4 | 77.2 | 2.7 |
| IS | 98.9 | 2.7 | 82.2 | 4.1 |

Table S7 Stability data of the KME in rat plasma under different conditions (n=6)

| Spiked  Concentration  (ng/mL) | Short-term | | Three freeze-thaw | | Post-preparative | |
| --- | --- | --- | --- | --- | --- | --- |
|  | Measured  (ng/mL) | RE  (%) | Measured  (ng/mL) | RE  (%) | Measured  ng/mL | RE  (%) |
| 1 | 0.92±0.04 | -8 | 1.03±0.07 | 3 | 1.04±0.09 | 4 |
| 100 | 98.2±2.99 | -1.8 | 113.0±4.47 | 11.3 | 100.3±2.15 | 0.3 |
| 400 | 415.6±12.93 | 3.9 | 412.2±46.71 | 3.1 | 387.0±21.30 | -3.3 |
